# Assessment of *Lactococcus Cremoris* preparations for the pre- and post-milking teat disinfection

**DOI:** 10.1186/s12917-024-04290-7

**Published:** 2024-09-28

**Authors:** A. Gazzola, M. Zucali, M. F. Addis, L. Bava, S. Morandi, S. Pisanu, D. Pagnozzi, A. Passera, M. Brasca, R. Piccinini

**Affiliations:** 1 Istituto Zooprofilattico Sperimentale della Lombardia e dell’Emilia-Romagna, Lodi, 26900 Italy; 2 Dept. Agricultural and Environmental Sciences, University of Milan, Milan, 20133 Italy; 3 Dept. Veterinary Medicine and Animal Science, University of Milan, Lodi, 26900 Italy; 4 Institute of Sciences of Food Production (ISPA), Italian National Research Council (CNR), Milan, 20133 Italy; 5 Porto Conte Ricerche, S.P. 55 Porto Conte-Capo Caccia, Km 8.400, Tramariglio, Alghero (SS), Italy; 6 Dept. Veterinary Medicine and Animal Science, via dell’Università 6, Lodi, 26900 USA

**Keywords:** Mastitis, Dipping, Nisin, Somatic cell count, Bacteriological analysis

## Abstract

Good hygienic milking practices including the disinfection of the cow teat skin before and after milking aimed at preventing the occurrence of new intramammary infections (IMI) in dairy cows. This practice is generally performed using disinfectants, and in the current context of attention to the emergence of resistances, it is of greatest interest to evaluate alternative solutions that can expand treatment options. We assessed the efficacy of a pre-dipping and a post-dipping product based on the bacteriocin-containing culture of Lactococcus cremoris strain FT27, as compared to commercial disinfectants. FT27 was selected for the bactericidal activity in vitro against food pathogens. In the present study, it also revealed a high bactericidal activity against the main mastitis pathogens, most likely related to Nisin A production, according to genomic and proteomic analysis. The lactococcus-based preparations were applied in two commercial dairy farms in Northern Italy. Overall, 298 dairy cows were enrolled, 141 in the treated group (TR), and 157 in the control group (CTR). The cows were sampled at quarter level every two weeks for 3 months. During the trial, all cows showed a good health status. The hygiene level for udder, flanks and legs was generally good (on average < 3 score); the udder hygiene was significantly worse (*P* < 0.01) in the CTR group. The teat apex hyperkeratosis was overall low (on average < 2 score). We recorded no significant differences between the two experimental groups in the Somatic Cell Count (SCC) values and the bacteriological results. The overall frequency of new IMI was low, ranging 0.6 − 0.5% for S. aureus in the TR or CTR group respectively, to 2.6–4.4% for NASM. Regarding Str. spp., the new IMI accounted for 1.7% or 1.9% in the TR or CTR group, respectively. Notably, the incidence of S. aureus new IMI did not increase during the study, even though neither of the two herds segregated the positive cows. The non-inferiority test showed that the Lactococcus-based pre- and post-dipping products had an efficacy comparable to proven commercial disinfectants in maintaining udder health and preventing new IMI, thus representing a possible alternative to current teat dip products.

**Supplementary Information:**

The online version contains supplementary material available at 10.1186/s12917-024-04290-7.

## Background

 Bovine mastitis is the most important disease of dairy cattle worldwide and a major cause of significant economic losses for the dairy industry. The application of proper hygienic milking practices is of primary importance for preventing new intramammary infections (IMI) in dairy cows [1]. These practices include the disinfection of the teat skin before and after milking, to reduce the colonization of the udder by pathogens through the teat canal. Since the teat apex represents the first physical barrier to the entry of mastitis pathogens, both udder hygiene and teat skin condition influence the establishment of new IMI [2, 3]. In fact, damages of the teat ends, such as roughness, dryness or hyperkeratosis due to mechanical trauma during the milking or to the applied teat tip products, increase the risk of bacterial colonization and facilitate the mastitis pathogens to enter the teat canal [3]. Thus, the pre- and post-dipping agents should reduce the pathogen load and maintain the teat skin healthy. Pre- and post-dipping are usually performed using chemical substances such as chlorhexidine, chlorine, iodine and iodophor solutions, or quaternary ammonium compounds. These substances, depending on the concentration and the method of application, may contribute to increase the levels of residues in milk, representing a concern for human health and a problem for the dairy industry [4, 5]. Also, different studies reported that the use of chemical disinfectants, and their consequent release into the environment, increase the risk of resistance not only to these compounds but also to antimicrobials [6–8]. Finally, the use of these compounds might influence the milk microbiota biodiversity, with an impact on animal health [9]. To address these problems, it is important to increase the range of available teat dip products. A promising alternative approach involves the use of bacteriocins. Bacteriocins are small molecules with antimicrobial properties produced by bacteria for competition and defense against other microorganisms, especially closely related species [10]. Lactic acid bacteria (LAB) are natural inhabitants of the intestinal tract of ruminants and are considered the best source of bacteriocins, because they have no adverse effects on animal and human health. Among LAB, *L. cremoris* is extensively used in the production of dairy products, particularly cheeses, Moreover, it is being used for its probiotic features, in pharmaceutical applications, and in biotechnological processes [11].

Some *Lactococcus* strains can produce nisin, a bacteriocin approved by the Food and Drug Administration (FDA) and the European Union for cheese production [12].

The bactericidal activity of these molecules has been tested in vitro, showing their efficacy especially against Gram-positive bacteria [13, 14]. Thus, bacteriocins have been proposed as disinfectants or teat dip products during the milking procedure, in combination or replacement of commercial teat dip solutions [15, 16].

Our research included an initial in vitro study aimed at: (a) evaluating the bactericidal activity of a strain of *Lactococcus cremoris* strain FT27 isolated from a goat cheese, against the main mastitis pathogens, and (b) characterizing the FT27 strain using genomics and proteomics. Thereafter, an in vivo study was performed, in order to investigate the efficacy of a pre- and post-dipping preparation containing the FT27 strain, as compared to a commercial iodine-based disinfectant.

## Results

### In vitro study. Characterization of L. Cremoris FT27: antimicrobial activity, genomics and proteomics

Based on microplate assays, the minimal inhibitory concentration (MIC) value, expressed as AU/mL, was the highest for the 3 different *Str. agalactiae* strains with a value of 128; for 2 *Str. uberis* strains the MIC was 16 and 32, respectively; for 2 *Str. dysgalactiae* strains the MIC was 4 and 8; for 2 *Ent. faecalis* and 2 *Staph. aureus* the MIC was 4. In all cases, the minimal bactericidal concentration (MBC) values were identical to the MIC values.

The genome size of *L. cremoris* FT27 was 2’784’916 bp with a GC content of 35.6%. The obtained contigs were 118, with L50 of 7 and N50 of 145’551. The Number of RNAs was 62 and the protein coding sequences obtained were 2’953, including 1’934 non-hypothetical and 1’019 hypothetical sequences, respectively. Genome density was 1.06 CDS/Kb.

Upon analysis of the MS/MS spectra generated by shotgun proteomics of the FT27 culture supernatant, the public *Lactococcus* spp. database returned 663 protein identifications (supplementary file, data sheet A; DOI: 10.17632/876d526878.1). Upon interrogation of the annotated FT27 sequence database, we obtained 16 additional protein identifications (supplementary file, data sheet B; DOI: 10.17632/876d526878.1). All identified proteins were subjected to Gene Ontology for the definition of biological functions (supplementary file, data sheet C; DOI: 10.17632/876d526878.1), enabling to pinpoint 8 proteins with antimicrobial activity or belonging to related pathways. Adding to the lantibiotic Nisin (NisA), the following components of its production, release, and regulation pathway were identified: NisB, NisE, NisI, NisK, NisP, NisR, and the ATP-binding subunit of the lantibiotic protection ABC transporter.

### In vivo study. Milk production, hygiene score and teat apex score

During the 3-month trial, a total of 298 dairy cows were sampled within the two herds: 67 belonged to the TR group and 75 to the CTR group in herd B, 74 cows to the TR group and 82 to the CTR in herd M.

The individual milk production, corrected for fat content (4%), was equivalent in all cows, with 36.6–38.6 kg/day in the CTR or TR group, respectively. TR cows had a significantly lower mean lactation number than CTR cows (2.17 vs. 2.3, *P* = 0.01). The udder hygiene score was also lower in TR than CTR cows (2.29 vs. 2.68, *P* < 0.01), while no differences were detected for flank and leg hygiene scores. The teat apex scores showed low mean values, always below 2. However, the scores of the front quarters were slightly higher than the hind quarters (median values 1.84 vs. 1.83 in the TR group; 1.88 vs. 1.7 in the CTR group). The only significant difference between groups was observed for the rear left quarters, which were statistically worse in the TR cows (1.67 vs. 1.87, *P* = 0.02).

### Somatic cell counts and bacteriological analysis

**Fig. 1:**
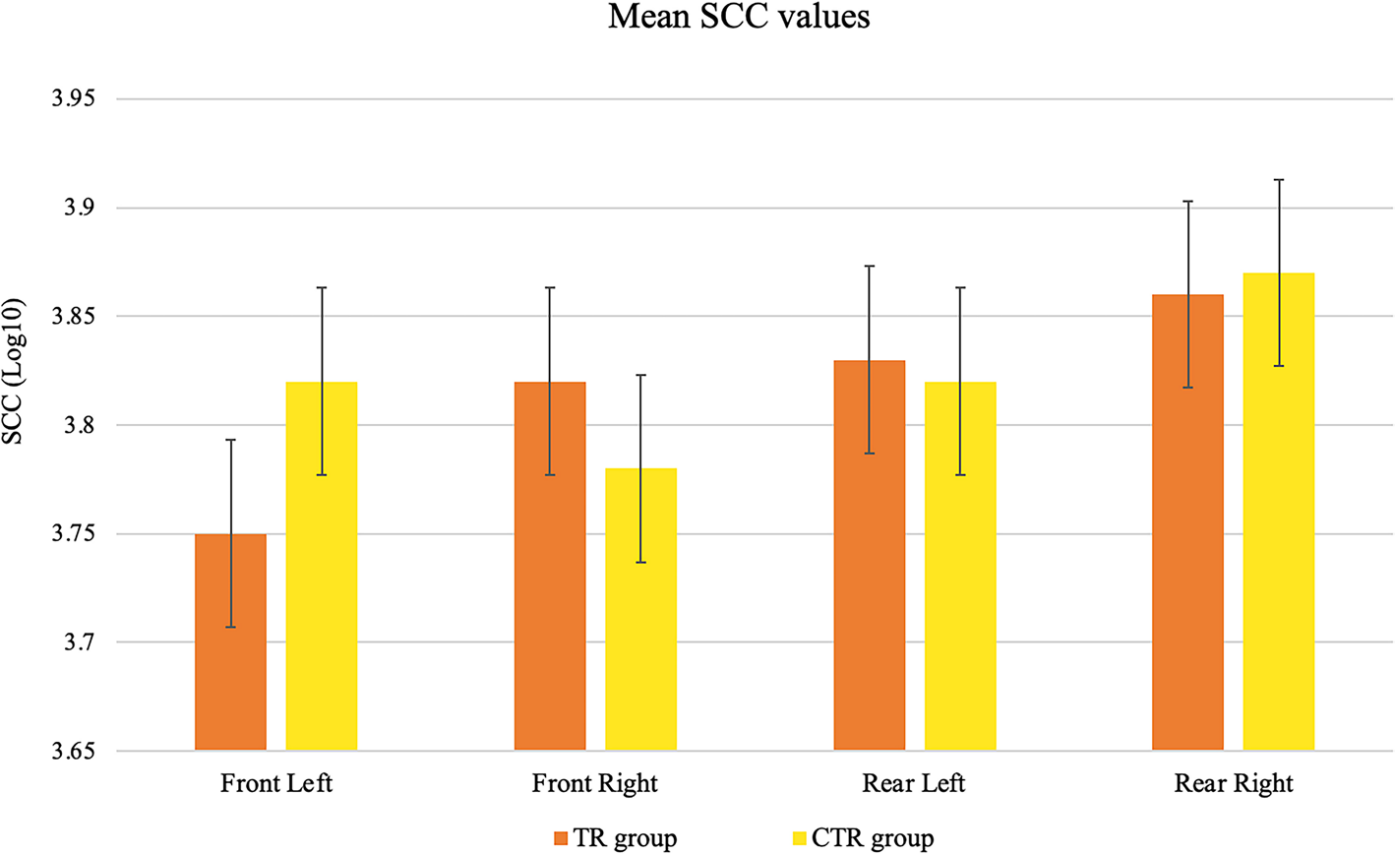
Mean Somatic Cell Counts (SCC, Log_10_ cells/mL) of single quarters from the *Lactococcus*-based dipping group (TR group) and control group (CTR group) collected during all the six samplings. The error bars indicate standard deviation

**Fig. 2:**
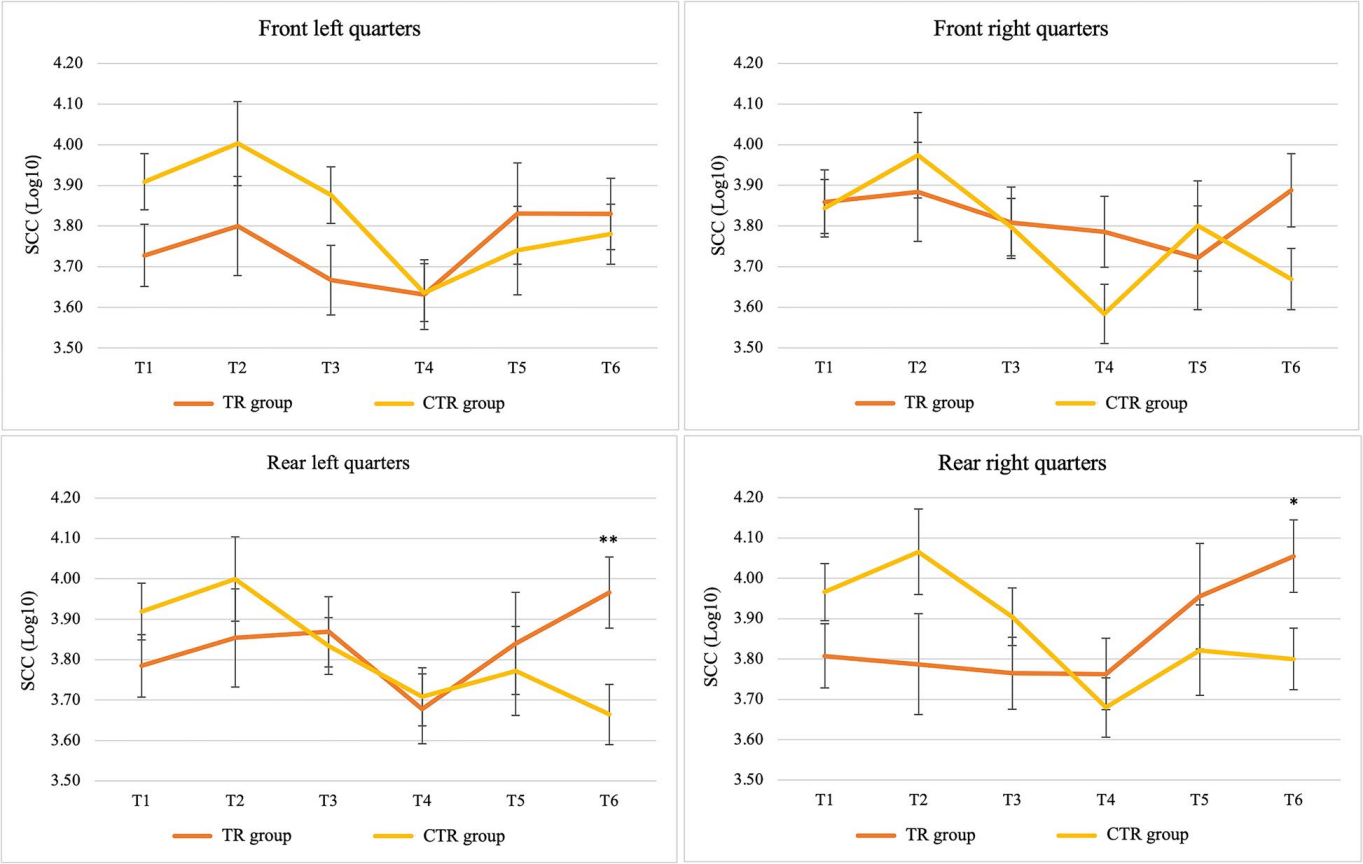
Somatic Cell Counts (SCC, Log_10_ cells/mL) of single quarters from the *Lactococcus*-based teat dip treated group (TR) and control group (CTR) throughout the study period. The bars indicate standard deviation. * Significance at *P* < 0.01. ** Significance at *P* < 0.05

Both dairy herds showed low SCCs throughout the study period, with mean values of 3.75 and 3.86 Log_10_ cells/mL (Fig. 1). In both TR and CTR groups, the SCCs decreased from the beginning to the end of the experiment, with the lowest values achieved at T4 in both groups. In the first two months, the quarters treated with the *Lactococcus*-based products showed lower SCC in comparison with the iodine dipped quarters, even if the difference was not significant, while a significant increase could be observed in both rear quarters of the *Lactococcus* group at the end of the study (Fig. 2).

**Fig. 3:**
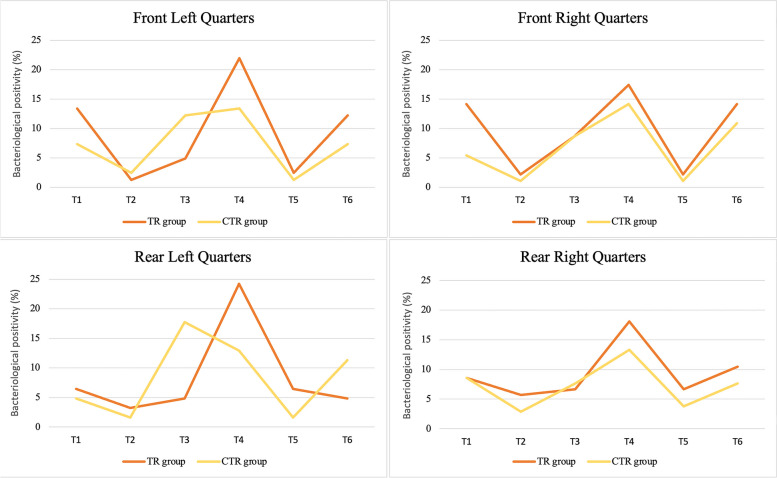
Frequency of bacteriological positive results of quarter milk samples from the *Lactococcus*-based teat dip treated group (TR, blue) and control group (CTR, orange) throughout the study period

The overall frequency of bacteriologically positive quarters was low and was not related to any SCC increase (Fig. 3). Changes in bacteriological positivities were observed in both groups during the trial, with a drop in the bacteriologically positive frequencies at T2, followed by an increase, a new drop and a final increase. The only difference between experimental and control group was evidenced at T6 in the rear left quarters. Interestingly, the quarters disinfected with the *Lactococcus*-based products showed a further decrease in bacteriological positivity, as opposed to the homologous iodine-dipped quarters.

For the statistical analysis, the isolates were divided into 4 categories: (1) contagious pathogens; (2) non-*aureus* staphylococci and mammalicocci (NASM); (3) environmental streptococci; (4) “other” environmental microorganisms, including Gram-negative bacteria and yeasts. The most frequent isolates were NASM (20% vs. 18.4% in the TR and CTR groups, respectively). Streptococci accounted for 13.6% and 11.1% of the positive quarter milk samples in the TR and the CTR groups, respectively, identified mainly as *Str. uberis* and *Str. dysgalactiae*. Among NASM, the most frequently detected were *Staph. chromogenes*, *Staph. epidermidis*, and *Staph. haemolyticus*. *Staph. aureus* was the only contagious pathogen detected and it was isolated in both herds, in 16 cows of the TR group and 12 cows of the CTR group. In most cases, it was present in one quarter at one or two time points.

**Fig. 4:**
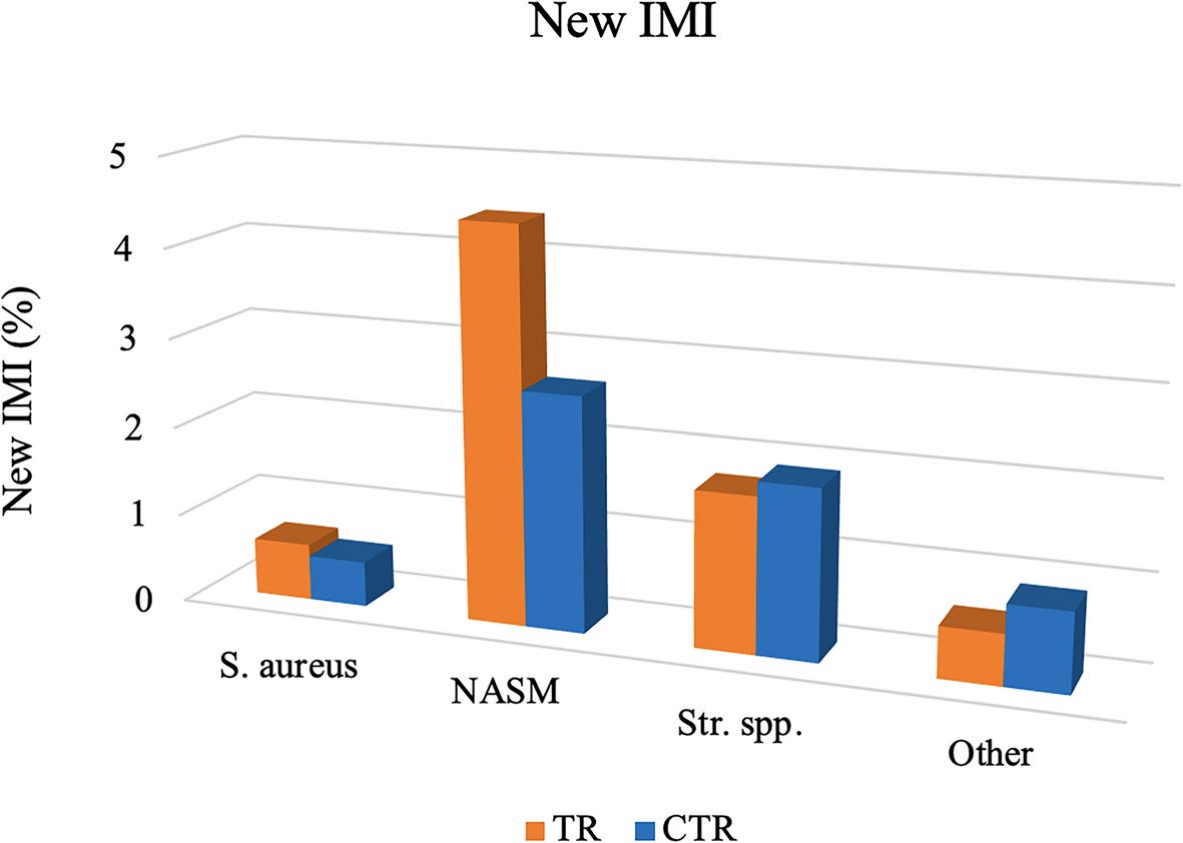
Total new IMI for bacterial species in quarter milk samples from the *Lactococcus*-based teat dip treated group (TR) and control group (CTR), collected during all the six samplings

The proportion of new IMI was very low, ranging 0.5–0.6% for *S. aureus* in the CTR or TR group respectively, to 2.6–4.4% for NASM. Regarding *Str. spp.*, the new IMI were on average lower in the TR group when compared with the CTR (1.7% vs. 1.9%; Fig. 4).

**Fig. 5:**
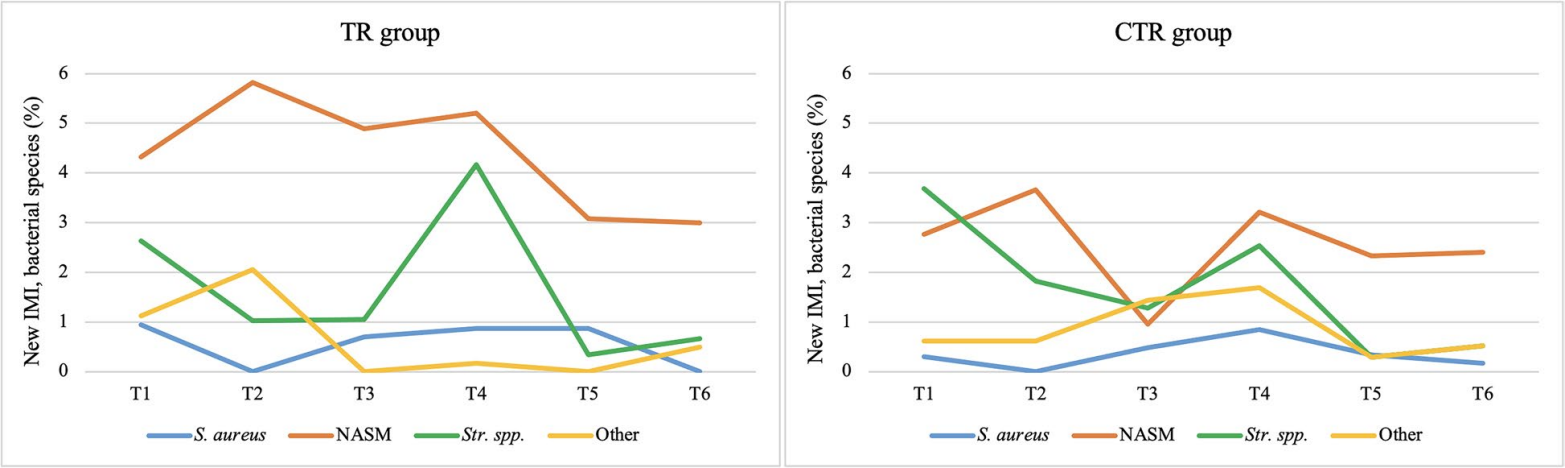
New IMI for bacterial species in quarter milk samples from the *Lactococcus*-based teat dip treated group (TR, left) and control group (CTR, right) throughout the study period (T1 to T6)

The pattern of the pathogens causing new IMI, isolated in the different time points, showed some differences among the four bacterial categories considered and between the TR and the CTR group (Fig. 5). NASM new IMI were always higher in the TR group, but decreased from the first to the last sampling, while in the CTR group the initial frequency was comparable to that detected at the end of the experiment. *S. aureus* and *Str. spp.* new IMI were similar in both experimental groups, but the frequency of the first pathogen showed only slight changes during the study, while *Str. spp*. decreased from T1 to T6, despite a peak observed at T4. The new IMI caused by Gram-negative bacteria were always lower in the TR group when compared to the CTR.

The Non-inferiority test showed that the efficacy of the experimental formulations was not inferior to the commercial iodine product (*P* < 0.001). The significance of the test was verified by the lower limit of the 95% confidence interval (0.0099) being greater than the non-inferiority limit (-0.035).

## Discussion

The pre- and post-milking disinfection of the teat is recommended to reduce bacterial load on the skin and still represents one of the most important practices to reduce the incidence of new IMI in dairy herds [1]. Moreover, the risk for microorganisms of developing biocide resistance, and potential cross-resistance to antibiotics, has been demonstrated after exposure to sub-inhibitory concentrations of antiseptics, disinfectants, and preservatives [17]. It has been demonstrated that over intrinsically Quaternary Ammonium Compounds (QAC)-tolerant microorganism, staphylococci can acquire resistance to these disinfectants together with antibiotic-resistance [18]. These phenomena highlight the importance of increasing the range of available teat dip products. For this reason, we investigated the efficacy of one formulation for the pre-dipping and a second one for the post-dipping, containing the nisin A-producing strain *L. cremoris* FT27 as compared to a iodine-based commercial product.

Bacteriocins have long been known and their antimicrobial activity has been largely demonstrated; however, little information is reported in the literature about the in vivo effectiveness of these proteins in preventing the spread of IMI in dairy cow herds. Moreover, the studies are usually conducted on a limited number of animals, which are sampled once or followed for just a few days. Both the administration of teat dipping formulations containing bacteriocin or bacteriocin-producing probiotics have been reported. Two different studies [15, 19] evaluated the reduction of mastitis-causing pathogens on the teat skin of 8 or 12 dairy cows, respectively, after dipping with a formulation containing either lacticin 3147, or bactofencin A, nisin and reuterin. In both cases, the counts of Gram-positive bacteria were reduced by the application of bacteriocins. Similar results were obtained by Yu et al. [16] by applying a formulation containing two probiotic strains of *Lactobacillus plantarum* in the pre- and post-dipping on 25 lactating cows for 10 days.

The culture supernatant of *L. cremoris* FT27 demonstrated pronounced in vitro bactericidal effects when tested against streptococci, including *Strep. agalactiae*, staphylococci, including *Staph. aureus*, and *E. coli* isolated from bovine mastitis. Genomic and proteomic analysis confirmed that the bacteriocin responsible for the pronounced FT27 antimicrobial activity against the main mastitis pathogens, observed up to a dilution of 1:128 of the cell-free overnight culture, was Nisin A. Shotgun proteomics enabled the detection of several components of the Nisin A production pathway, including NisA, NisB, NisE, NisI, NisK, NisP, NisR, and the ATP-binding subunit of the ABC-transporter, which are responsible for the production and release of nisin in *L. lactis* [20]. Therefore, the pronounced antimicrobial activity of the FT27 strain compared to other *Lactococcus* strains might be related to higher production levels or longer half-life on Nisin A rather than the production of alternative or additional bacteriocins. Nisin A, a type-A lantibiotic, is one of the most studied bacteriocins [21]. Nisin A is recognized as safe and already approved and used as food additive in different human food categories, including dairy products such as cheese, thanks to its antimicrobial activity [22]. It successfully inhibits the growth of a wide range of Gram-positive bacteria with a duplicate mechanism consisting of pore formation in the bacterial membrane in combination with inhibition by peptidoglycan synthesis [23].

Our study lasted 3 months and enrolled 298 dairy cows in two commercial dairy farms characterized by a good udder health status, as highlighted by the low milk SCC. The individual milk production, corrected for fat content (4%), was higher in both treated and control group, in comparison with the values reported by the National Breeding Association (AIA, 2022). The hygiene level for udder, flank and legs was generally good, as shown by the hygiene scores; the only significant difference between the experimental groups regarded the udder hygiene, which was worse in the CTR group even if the barn management did not differ between the cow groups. The teat apex hyperkeratosis was overall low, but front quarters showed slightly higher values, in accordance with Gleeson et al. [24], due to the lower production level of front quarters and the following higher frequency of overmilking events.

During the study, we did not record significant differences between the control and the treated group in the SCC values or in the bacteriological results, highlighting the efficacy of the two experimental *Lactococcus*-based formulations. A clear demonstration of such efficacy was given by the Non-inferiority test, which was statistically significant. These results confirm our in vitro findings and expand the in vivo results of previous studies [15, 16, 19] indicating the effectiveness of bacteriocins in the prevention of dairy cow IMI. However, the efficacy of lactococci teat dipping on udder health in herds with low somatic cell counts may not reflect its effectiveness in higher challenge scenarios, such as herds with elevated milk somatic cell counts.

*Staph. aureus* was the only contagious mastitis pathogen, and it was isolated in both the control and the treated group, but only in a few udder quarters. Interestingly, the incidence of this pathogen did not increase during the experimental period, even though neither of the two herds segregated the positive cows, thus confirming the antimicrobial activity and efficacy not only of the commercial disinfectants but also of the *Lactococcus*-based products. Also the frequency of all the other mastitis-causing pathogens did not substantially differ between the treated and control group, further demonstrating the good activity of the experimental *Lactococcus*-based formulations containing nisin.

## Conclusions

The use of the *Lactococcus*-based preparation for the pre- and post-dipping has shown comparable efficacy to proven commercial disinfectants in preventing new IMI and maintaining udder health, thus representing a possible alternative to current teat dip products based on chemical substances. Furthermore, the use of formulas containing nisin-producing *L. cremoris* strains for the teat disinfection can open interesting perspectives for the conversion of waste products of the food industry into by-product, starting from the potential use of culture broth following the removal of viable cells, creating a simple production process based on production scraps.

## Methods

### In vitro *study*

#### Bacterial strain and growth condition

*L. cremoris* FT27 (isolated from a goat cheese and deposited in the Agro-Food Microbial Collection of the Institute of Sciences of Food Production, CNR, Bari, Italy as ITEM, 18332) was selected for this study based on previous evidence of antimicrobial activity against food pathogens due to bacteriocin or bacteriocin-like compound production [25]. The FT27 strain was routinely propagated aerobically in de Man, Rogosa, and Sharpe (MRS) broth (Scharlau Microbiology, Barcelona, Spain) overnight at 30 °C, and was preserved in Litmus milk (Biolife Italiana, Milan, Italy) at − 18 °C.

#### *L. Cremoris* FT 27 characterization: antimicrobial activity, genomics and proteomics

The FT27 strain was tested in vitro against the main mastitis-causing microorganisms (*Staph. aureus* MB 535, 543; *Strep. agalactiae* MB 90, 98, 386; *Str. uberis* MB 705, 707; *Str. dysgalactiae* MB 280, 324; *Enterococcus faecalis* MB 561, 706), using the MIC and MBC assay described by Malvisi et al. [26]. The tests were performed in triplicate and repeated twice on different *L. cremoris* FT27 cultures. The reference strains belonged to the Laboratory collection and had been isolated from bovine clinical or subclinical mastitis.

Genomic DNA of the FT27 strain was extracted from overnight cultures using the Dneasy UltraClean Microbial kit (Qiagen GmbH, Hilden, Germany) following the manufacturer’s instructions. Yield and purity of DNA were evaluated using the Infinite F200 PRO microplate reader (Tecan, Mannedorf, Switzerland). The extracted DNA was stored at − 20 °C and later shipped to the sequencing service facility Personal Genomics S.r.l. (Verona, Italy) for libraries’ preparation and sequencing and whole-genome DNA was sequenced through the Illumina MiSeq platform. Gene annotation was performed using RAST web service [27].

For proteomic characterization, six 1 mL aliquots of an overnight culture of *L. cremoris* FT27 were centrifuged for 10 min at 9,300 x g in a refrigerated Eppendorf microcentrifuge. The supernatant was collected and concentrated in Amicon Ultra devices with a molecular cutoff of 3 kDa and 10 kDa. The four fractions obtained (< 3kDa, < 10 kDa, > 3 kDa, and > 10 kDa, respectively) were assessed for antimicrobial activity as described above after reconstituting the concentrated retentate of each sample (fraction with molecular weight (MW) < 3 kDa and < 10 kDa, respectively) to the original volume of 1 mL with mQ water. Since the antimicrobial activity was located in the fraction > 10 kDa, two technical replicates of this fraction were subjected to a proteomics analysis pipeline. Peptides for mass spectrometry analysis were generated by on-filter reduction, alkylation, and trypsin digestion with the filter‐aided sample preparation procedure [28], with minor modifications [29]. Shotgun proteomic analysis was carried out by tandem mass spectrometry on a LTQ-Orbitrap Velos (Thermo Fisher Scientific, San Jose, CA, USA) paired with an UltiMate 300 RSLnanoLC system (Thermo Scientific, CA, USA), as described by Pisanu et al. [30]. Briefly, run analysis was performed loading a total of 4 µg of peptide mixture using a linear gradient of 245 min. The peptide fraction was concentrated, washed, and separated onto a trapping precolumn (Acclaim PepMap C18, 75 μm × 2 cm nanoViper, 3 μm, 100 Å, Thermo Scientific) and a C18 reverse-phase column (Easy-Spray PepMap C18, 75 μm × 50 cm nanoViper, 100 Å, Thermo Scientific), respectively. Tandem mass spectrometry analysis was performed in a data-dependent MS/MS mode using Higher Energy Collision (HCD) as a fragmentation method and nitrogen as the collision gas. Protein identification was carried out by Proteome Discoverer (version 2.4; Thermo Scientific) as described previously [30]. Raw files were analyzed against the *Lactococcus* spp. database (UniptroKb, release_2021_04) and against the custom database generated upon sequencing and annotation of FT27 genome. Gene ontology analysis was carried out with UniProt Knowledgebase (UniProtKB) database to obtain protein annotations for biological processes, molecular functions, and cellular components.

#### Preparation of pre- and post-dipping products

The basic components of the two experimental products were the same as those used in commercial formulations by the Company supporting the present study. The pre-dipping product contained surfactants and foaming agent, while the post-dipping included emollients, skin protective components, and film-forming substances. The FT27 strain was grown overnight in MRS broth and the culture was diluted 1:4 to the basic components of both the pre- and the post-dipping formulations, following MIC and MBC results. After adding the basic components, both pre- and post-dipping formulations were tested for FT27 viability and the strain did not grow on the MRS agar. Therefore, in order to limit time-consuming and costly procedures, we decided to avoid the centrifugation of *L. cremoris* broth cultures. For each herd, the bacteriocin-containing broth culture was produced once at the beginning of the trial, because the results of MIC tests, run over 15 weeks prior to the beginning of the field trial, demonstrated that the product remained active and stable for more than 3 months. Bacteriocin content, determined in the fresh culture according to Pongtharangkul & Demirci (2004) [32], was about 950 IU/mL.

The commercial pre-dipping product contained lactic acid, while the post-dipping a high concentration of iodine (3,000 ppm).

### In vivo *study*

#### Herds and experimental design

The study was carried out on two farms breeding Friesian dairy cattle, located in the Lombardy Region (Italy). At the time of the study, the herd dimension was 160 and 210 lactating cows, respectively for farm B and M. The average individual daily production level was 31.4 ± 8.44 kg for farm B, and 36.7 ± 8.73 for farm M. Milk somatic cells count (SCC) was 2.44 ± 1.87 log_10_ cells/ml for farm B, and slightly lower for farm M (1.98 ± 1.78 log_10_ cells/ml).

Both farms have been carrying out a complete milking routine, which consisted of disinfecting the cow teats with a pre-dipping product, drying the teats with disposable service paper towels, forestripping and applying a post-dipping product right after the teat cup removal. In both herds, the lactating cows were divided into a “control group” (CTR) and a “treated group” (TR) so as to balance the overall health status and characteristics of the two experimental groups within each herd. The commercial teat dip products were applied to the cow teats in the CTR group and the experimental preparations to the TR group. The cows under antibiotic treatment were excluded from the study. Overall, 141 cows were enrolled for using *Lactococcus*-based pre- and post-dipping products (TR), and 157 for dipping with commercial formulations (CTR). In both herds, during the 3-month trial, six samplings were carried out from February to April, every two weeks following the same protocol (T1-T6).

Before the beginning of the experiment, to evaluate the udder health status, all the lactating cows were sampled twice, by collecting quarter milk samples for the bacteriological analysis and the SCC evaluation. Three days after the second sampling, since no important differences had been observed between the two groups of cows, and the prevalence of contagious pathogens was lower than 5%, the experimental trial started with the application of the above-mentioned dipping products.

#### Milk sampling, teat, and hygiene scoring

After discarding the first streams of milk, samples from each single udder quarter of the cows belonging to the two experimental groups were aseptically collected in 10 ml tubes, according to National Mastitis Council guidelines [33] and immediately stored at 4 °C. During the samplings, the assessment of the integrity of the teat apex was also carried out on all the sampled cows, with the evaluation model of the *Teat apex score* [34], and the evaluation of the state of soiling of the flanks, legs and udder, using the scheme of the *Hygiene Score* [35]. Blind evaluations were performed by two trained experts during farm visits.

#### Bacteriological analysis and SCC

After collection, the samples were kept at 4 °C and analyzed within 24 h. Ten microliters of milk were plated on blood agar plates supplemented with 5% defibrinated bovine blood, incubated aerobically at 37˚C, and analyzed after 24 and 48 h, according to the National Mastitis Council guidelines [36]. The colonies were identified using the matrix-assisted laser desorption/ionization time-of-flight mass spectrometry (MALDI-TOF MS). The mass spectra were acquired with the MBT Microflex LT/SH MALDI-TOF mass spectrometer (Bruker Daltonik GmbH) in the positive mode and interpreted against the MBT Compass^®^ 4.1 database. For the calibration of the instrument, the Bacterial Test Standard (Bruker Daltonik GmbH) was included in each target plate. A quarter was defined as infected (IMI) if one or two different bacterial species were identified; in the presence of more species, the milk sample was defined as contaminated [36].

The SCC was determined using a Bentley Somacount 150, which is based on the flow cytometry methodology for somatic cell counting (Bentley Instrument, Chaska, MN, USA).

### Statistical analysis

The data were analyzed using SAS 9.4 (2012). Descriptive statistics were perfomed using FREQ and MEAN procedures.

#### Definition of New IMI

An IMI was considered as a new IMI when a different microbial species was detected in a single quarter milk sample that was not present, in that quarter, in the previous control. Afterwards, only a new bacterial species, not previously identified in that quarter, was eligible to cause a second or third new IMI. The incidence of new IMI during the study period was calculated as the number of quarters with a new IMI, divided by the amount of quarters analysed across all time points.

#### Non-inferiority test

We used the difference in the proportion of new IMI to evaluate non-inferiority of the experimental product, by the means of the Wald test, with 95% confidence intervals (CI). We applied the same non-inferiority margin (3.5%) used by Godden et al. [37].

#### Sample size calculation

Assuming a 3.5% new IMI rate (d = 0.035) for the TR group, it was estimated that at least 478 quarters per treatment group were required to provide the desired 80% power and 95% confidence (α = 0.05), in order to detect a treatment difference, if truly present. In the present study, the sample size was bigger than the requested.

#### Effect of treatment

For testing the effects of *Lactococcus*-based preparations, the data collected during the study were subjected to analysis of variance using a Global Linear Model. The model used was:

$${\mathrm Y}_{\mathrm{ijkl}}\;=\;\mathrm\mu+{\mathrm R}_{\mathrm i}+{\mathrm S}_{\mathrm j}+{\mathrm{RS}}_{\mathrm{ij}}+{\mathrm P}_{\mathrm k}+{\mathrm L}_1+{\mathrm F}_{\mathrm m}+\mathrm C+{\mathrm e}_{\mathrm{ijklmn}\;}$$

Where Y_ijklmn_=dependent variables; µ = general mean; R_i_= *Lactococcus*-based teat dip effect (i = CTR; TR), S_j_=the date of the sampling (j = T1-T6), RS_ij_=the interaction between the group and the date of the sampling, P_k_=the number of lactations (k = 1-2-≥3), L_l_=the stage of lactation (l = < 100, 100–200, ≥ 200 Days in Milk), F_m_=farm effect (m = Farm B and M) and C = as covariate the values of somatic cells (composite milk samples) for the month of January, before the starting of the study (obtained from the controls carried out monthly on the farm by the National Breeding Association) were included in the model.

The dependent variables considered were Somatic Cell Counts (Log_10_ cells/mL) of single quarters, hygiene score, teat apex score, milk production.

## Supplementary Information

Supplementary Material 1.

## Data Availability

The Lactococcus cremoris FT27 strain used during the current study is deposited in the Agro-Food Microbial culture Collection of the Institute of Sciences of Food Production (CNR, Bari, Italy) as ITEM, 18332.All the protein identifications and their biological functions are available in the supplementary information file (DOI: 10.17632/876d526878.1, data sheet A, B, and C).

## Abbreviations

CTR: Control group
IMI: Intramammary Infection
MBC: Minimum Bactericidal Concentration
MIC: Minimum Inhibitory Concentration
NASM: non-aureus Staphylococci and Mammalicocci
SCC: Somatic Cell Count
TR: Treated group
